# Antitubercular Activity of the Fungus *Gliocladium* sp. MR41 Strain

**DOI:** 10.22037/ijpr.2019.1100667

**Published:** 2019

**Authors:** Andrés Humberto Uc-Cachón, Marcela Gamboa-Angulo, Rocío Borges-Argáez, Manuela Reyes-Estebanez, Salvador Said-Fernández, Gloria María Molina-Salinas

**Affiliations:** a *Unidad de Investigación Médica Yucatán, Unidad Médica de Alta Especialidad Hospital de Especialidades 1 Mérida, Yucatán, Instituto Mexicano del Seguro Social, México. *; b *Unidad de Biotecnología, Centro de Investigación Científica de Yucatán A.C., Mérida, Yucatán, México.*; c *Departamento de Microbiología Ambiental y Biotecnología, Universidad Autónoma de Campeche, San Francisco de Campeche, Campeche, México.*; d *Centro de Investigación Biomédica del Noreste, Delegación Nuevo León, Monterrey, Nuevo León, Instituto Mexicano del Seguro Social, México.*; e *Departamento de Bioquímica y Medicina Molecular, Facultad de Medicina, Universidad Autónoma de Nuevo León, Monterrey, Nuevo León, México.*

**Keywords:** Gliocladium, Mycobacterium tuberculosis, Antituberculosis activity, Ergosterol-5, 8-peroxide, Allitol

## Abstract

Tuberculosis (TB) is a leading cause of death worldwide from infectious diseases and its inadequate treatment has led to emergence of resistant strains. The emergence of these strains renders the search for new drugs for the treatment of TB. The aim of this study was the evaluation of the anti-TB activity of the extract from fungus *Gliocladium *sp. MR41, and bioassay-guided fractionation and identification of majority compounds was carried out. Fungal strain culture was lyophilized and extracted by maceration in Ethyl Acetate (EtOAc). This extract was fractionated by liquid-liquid partitioning and chromatographic techniques, and the compounds were identified by their spectroscopic data. Furthermore, the EtOAc extract, fractions, and pure compounds were tested on *Mycobacterium tuberculosis* using the Microplate Alamar Blue Assay. From the bioactive AcetoNitrile Fraction (AcNF; MIC = 3.13 µg/mL) of the EtOAc extract, four compounds were isolated: ergosterol (1), ergosterol-5, 8-peroxide (2), 1, 6-di-*O*-acetyl-2,3,4,5-tetrahydroxy-hexane (3), and allitol (4). Only 2 exhibited potent activities against *M. tuberculosis* (MIC = 0.78 µg/mL). Additionally, this is the first report, to our knowledge, of polyols 3 and 4 from this fungus.

## Introduction

Tuberculosis (TB) is an infectious disease caused by the bacillus *Mycobacterium tuberculosis*, which typically affects the lungs (pulmonary TB), but it can also affect other sites (extrapulmonary TB) (1). TB gives rise to poor health in millions of people each year, and in 2016, it was the ninth leading cause of death worldwide and the leading cause from a single infectious agent, ranking above HIV/AIDS. In 2016, there were an estimated 10.4 million new cases of TB and 1.3 million deaths in worldwide. Treatment lasts about 6 months with five first-line drugs (Streptomycin, Isoniazid, Rifampin, Ethambutol, and Pyrazinamide). Misuse of these drugs, in addition to inconsistent or partial treatment, has led to the development of multiDrug-Resistant (MDR) TB and eXtensively Drug-Resistant (XDR) TB (2). There is an urgent need for new anti-TB drugs. 

In recent years, a large number of fungal natural products has been reported to possess *in-vitro *antimycobacterial activity with different chemical structures. From endophytic fungus *Phomposis* sp. PSU-D15 was isolated phomoenamide, an enamide dimer with a Minimal Inhibitory Concentration (MIC) of 6.25 µg/mL (3), and from the PSU-N24 fungal strain was isolated 9-α-hydroxy-halorosellina A, a hydronapthalenone derivative (MIC = 12.5 µg/mL) (4). From the insect-pathogenic fungus *Aschersonia tubulata*, the triterpenes dustatin and 3β-acetoxy-15α, 22-dihydroxyhopane (MIC = 12.5 µg/mL) (5) were isolated. The most promising compounds from fungal sources are the diterpene pleuromutilin isolated from *Clitopilus passeckerianus* (formerly *Pleurotus passeckerianus* [MIC = 3.1 µg/mL]) (6), and the peptide nocathiacin I, isolated from *Nocardia* sp. (MIC = 0.008 µg/mL) (7). At present, these compounds are found in different clinical-trial phases (8, 9). 

The genus *Gliocladium *belongs to the Moniliaceae family and its species are saprophytic fungi that generally live on the ground, although they have also been found in association with plants and animals of aquatic environments (10). In recent times, species of this genus have received attention concerning their biological and chemical properties. Some compounds isolated from species of the genus *Gliocladium *have exhibited important biological activities, such as secalonic and heptelidic acids with cytotoxic and antibacterial activities, respectively (11, 12). From *Gliocladium *sp. FO-1513 were isolated the polyisoprene compounds glisoprenins A and B that inhibited acyl-CoA: cholesterol acyltransferase activity (13). In addition, from* G. roseum *KF-1040 were isolated some roselipins –structures of highly methylated C20 fatty acid, mannose, and arabinitol– that inhibitied diaciylglycerol acyltransferase activity (14). Recently, a diketopiperazine, alkaloid cyclo-(glycyl-L-tyrosyl)-4,4-dimethylallyl ether, was isolated from a strain of *Gliocladium *sp., and this compound exhibited strong antimicrobial activity against *Micrococcus luteus *(15). Sterol derivatives and anthraquinones were isolated from *G. catenulatum*, and these compounds inhibited the proliferation and growth of a myelogenous leukemia line (K562) (16). 

Our research groups reported the anti-TB activity of the Ethyl Acetate (EtOAc) extracts of three strains of the *Gliocladium* genus from Yucatán, México (17). In addition, another strain of *Gliocladium *sp. MR41, was isolated from leaf litter in Veracruz, México (18). In our present contribution, the extract from this fungal strain was tested *in-vitro* against *M. tuberculosis*, and bioassay-guided fractionation and the identification of majority compounds were carried out.

## Experimental

*General chemical experimental procedures*

Silica gel 60 (Merck, Darmstadt, Germany) or Sephadex LH-20 (Amersham Pharmacia Biotech AB, Uppsala, Sweden) was used for Column Chromatography (CC). Detection in Thin-Layer Chromatography (TLC) was achieved under Ultra Violet (UV) light and by spraying with the phosphomolybdic acid reagent, followed by heating for 5 min at 105 ºC. Melting point was determined on a Mel-Temp II. InfraRed (IR) spectra were recorded on KBr discs on a Nicolet Protégé 460. ^1^H-, while ^13^C-Nuclear Magnetic Resonance (NMR) spectra were recorded on a Bucker Avance 400 Ultra Shield spectrometer in CDCl_3_ or D_2_O, with Tetra Methyl Silane (TMS) as internal standard. EIMS were obtained on an Agilent Technologies 6890N gas chromatograph coupled with an Agilent Technologies 5975B intermass selective detector. 

*Fungal culture*

The strain of *Gliocladium *sp. MR41 was obtained from the culture collection of the Centro de Investigación Científica de Yucatán, previously identified by molecular taxonomy (18). This strain was reactivated on Petri dishes with corn agar and incubated during a 12/12-h light/dark photoperiod at 25 °C for about 7**–**8 days. Finally, the hyphae/spores of this fungus was obtained and inoculated onto fermented rice as substrate, as described previously (18), and this was incubated for 40 days at 25 °C with a 12/12-h light/dark photoperiod. At the end of the growth, the fungal cultures were frozen, lyophilized, and powdered.

*Extraction and Purification*

Several batches of the ground fungal material were extracted by maceration in EtOAc (3×) at room temperature. The solvent was filtered and evaporated *in vacuo* to yield a dry EtOAc extract (8.0 g). This extract was fractionated by liquid-liquid partition, with *n*-hexane and acetonitrile (3×, 2:1, 1:1, and 1:1, *v*/*v*), and both layers were evaporated under reduced pressure to produce the Hexanic (HexF) and AcetoNitrile Fractions (AcNF). During the evaporation of AcNF, a precipitate was obtained and purified by crystallization with acetone to yield compound **1** (230 mg). The AcNF (510 mg) was subjected to CC on Sephadex LH-20 and eluted with MeOH to yield six fractions (F1–F6). The bioactive F6 (47.7 mg) was chromatographed by vacuum liquid chromatography on silica gel and eluted with *n*-hexane, *n*-hexane: CH_2_Cl_2_, CH_2_Cl_2_: MeOH, and MeOH as ascending polarity gradient to yield 18 subfractions (SF1–SF18) according to TLC analysis. The SF1 was purified by flash CC on silica gel using mixtures of *n*-hexane:EtOAc with gradient elution to yield compound **2** (4.0 mg). The SF9 was partitioned with EtOAc and water (1:3, *v*/*v*), and the aqueous layer was lyophilized to yield compound **3 **(11.5 mg). The SF15 was purified by successive crystallizations with acetone to yield compound **4 **(10.1 mg).

**Figure 1:**
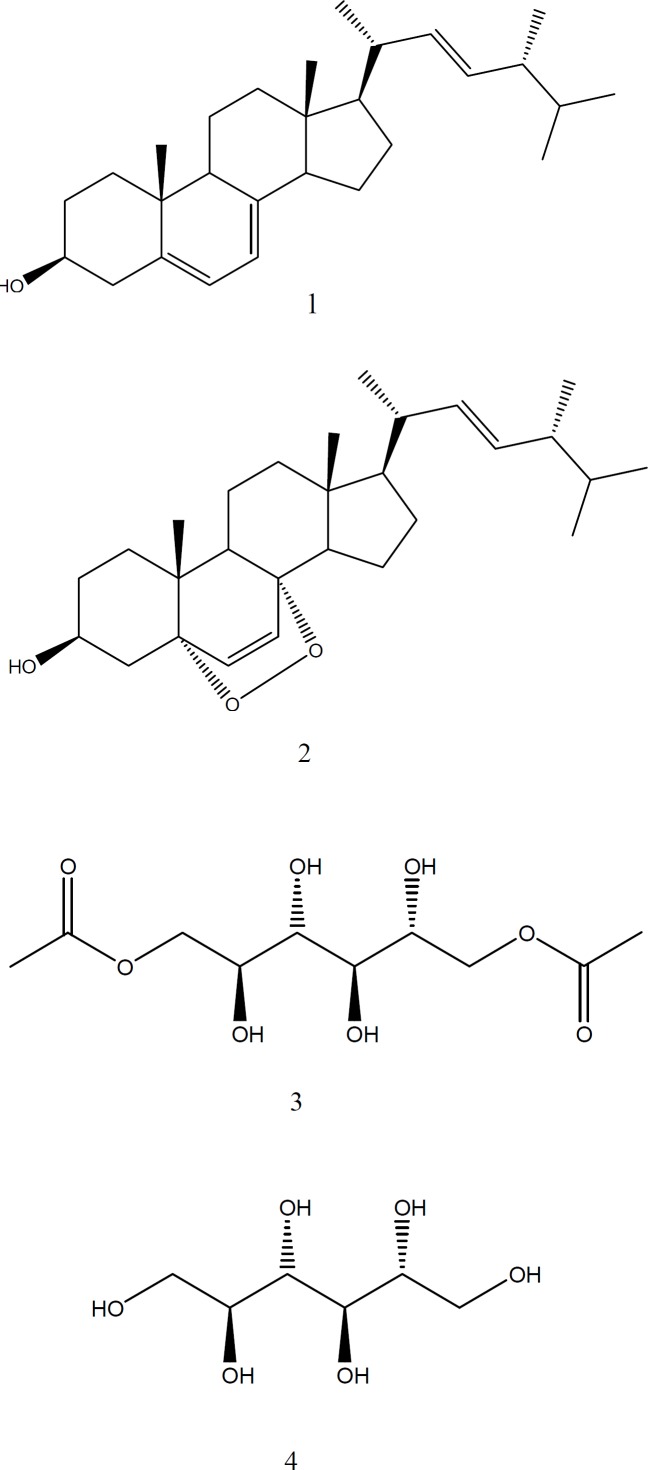
pounds isolated from *Gliocladium *sp. MR41. Ergosterol (1), ergosterol-5,8-peroxide (2), 1,6-di-*O*-acetyl-2,3,4,5- tetrahydroxy-hexane (3), and allitol (4)

**Table 1:** Anti-TB activity of extract, fractions, and compounds of *Gliocladium *sp. MR41.

**Crude extract/ fractions/compounds**	**MIC (µg/mL) on ** ***Mycobacterium tuberculosis *** **H37Rv**
EtOAc extract	6.25
HexF	>100
AcNF	3.13
F1	100
F2	100
F3	100
F4	100
F5	50
F6	6.25
Ergosterol (1)	>100
Ergosterol-5,8-peroxide (2)	0.78
6-di-*O*-acetyl-2,3,4,5-tetrahydroxy-hexane (3)	100
Allitol (4)	100
Rifampin	0.062

EtOAc: Ethyl AcetateHexF: *n*-Hexane Fraction; AcNF: AcetoNitrile Fraction; F: Fraction.

*Structural Identification of compounds*

1,6-di-*O*-acetyl-2,3,4,5-tetrahydroxy-hexane (**3**): white solid; [α]^D^_25 _= 0 (c = 0.014, MeOH); mp. 126-128 °C; IR ν_max_ (KBr) 3328 (O-H), 1743 (C=O), 1234 (C-(C=O)-C), 1078 (C-O) cm^-1^; ^1^H-NMR (400 MHz, CD_3_OD) δ 2.08 (CH_3-_COO), 3.79 (2H, d, *J *= 8.8 Hz, H2, H5), 3.87 (2H, m, H3, H4), 4.17 (2H, dd, *J *= 6.0 Hz, 11.5, H1a, 6a), 4.38 (1H, dd, *J *= 2.4, 11.5 Hz, H1b, 6b); ^13^C-NMR (125 MHz, CD_3_OD) δ 20.8 (CH_3-_COO), 67.9 (C1-C6), 70.1 (C2-C5), 70.4 (C3-C4), 173.2 (CH_3-_COO).

Allitol or 1,2,3,4,5,6-hexahydroxy-hexane (**4**): white solid; [α]^D^_25 _= 0 (c = 0.006, MeOH); mp. 154-156 °C; IR ν_max_ (KBr) 3282 (O-H), 1020-1083 (C-O) cm^-1^; ^1^H-NMR (400 MHz, CD_3_OD) δ 3.62 (2H, dd, *J *= 5.8, 10.9 Hz, H-1a, 6a), 3.69 (2H, m, H-2-5), 3.77 (2H, d, *J *= 7.8 Hz, H-3-4), 3.81 (2H, dd, *J *= 3.4, 10.9 Hz, H-1b,6b). 

*Antimycobacterial assay*

The *in-vitro *assay was assessed on the *M. tuberculosis *H37Rv strain (ATCC 27294) susceptible to all five first-line anti-TB drugs (Streptomycin, Isoniazid, Rifampin, Ethambutol, and Pyrazinamide). The microorganism was inoculated in 13 × 100-mm screw-capped tubes containing 3 mL of sterile Middlebrook 7H9 broth (Difco, Detroit MI, USA), supplemented with 0.2% glycerol and enriched with 10% Aleic acid, Albumin, Dextrose, and Catalase (OADC) (Difco) incubated at 37 °C in 5% CO_2_ atmosphere. Anti-TB activity was determined by the Microplate Alamar Blue Assay (MABA) described previously by Molina-Salinas *et al. *(19). The crude extract, fractions, or pure compounds were dissolved with DiMethyl SulfOxide (DMSO). All samples were tested using a concentration range of 100**–**0.39 µg/mL, and the maximal concentration of DMSO in the assays was 1.2% (v/v). MIC was defined as the lowest concentration of each sample that prevented the color change from blue to pink. In each microplate, 1.00**–**0.031 µg/mL of Rifampin was included as positive control. In addition, a blank (extract of fermented rice) and DMSO were included as negative and solvent controls, respectively. All evaluations were carried out in triplicate.

## Results

*Extraction and Purification*

The fungus *Gliocladium* sp. MR41 was massively cultured and the EtAcO crude extract was obtained. This extract was solvent-partitioned to obtain HexF and AcNF. The bioassay-guided fractionation of AcNF led to the obtaining of four pure compounds: **1–4 **(Figure 1).

*Structural Identification of compounds*

Compounds 1 and 2 were identified as ergosterol and its derivative, ergosterol-5,8-peroxide, respectively, according to their spectral data and by comparison with standard samples. In addition, two compounds were purified, which presented similar spectral data. Compound 3 presented an intense band of alcohol (3328 cm^-1^) and ester (1743 cm^-1^) groups. Compound 4 showed no carbonyl group and, and its ^1^H-NMR spectra showed signals typical of the oxygenated protons (3.62–3.81 ppm). These spectral data indicated a polyol structure; then, its melting point (154−156 °C) matched with that reported for a hexytol named allitol (4). On the other hand, the ester group of compound 3 was confirmed by their ^1^H and ^13^C NMR spectrum. These corresponded to two acetate groups (20.8 and 173.2 ppm), since it is a symmetrical structure and it correlated in all proton-spectra integrations. Therefore, compound **3** was identified as an ester derivative denominated 1,6-di-*O*-acetyl-allitol. Relative stereochemistry at positions C-4 and C-5 was established by the interaction observed on Two-Dimensional-Nuclear Overhauser Enhancement SpectroscopY (2D-NOESY), and this established a *trans *relationship between H-4 and H-5.

*Antitubercular activity*

The results of *in-vitro* anti-TB activity of the crude extract, fractions, and pure compounds are depicted in Table 1. Bioactivity was found in solvent-partition AcNF (MIC = 3.13 µg/mL) and its fractionation (F1−F6) showed that bioactivity remains in F6 (MIC = 6.25 µg/mL). Four compounds (1–4) were isolated from F6, and only compound **2** exhibited bioactivity (MIC = 0.78 μg/mL).

## Discussion

In the search for novel alternative natural products in the discovery and development of new active drugs against *M. tuberculosis*, our research group has been screening fungi isolated from several states of México. Among these, a non-previously tested *Gliocladium* sp. MR41 strain revealed a remarkable anti-TB property (MIC = 6.25 μg/mL), justifying the bioassay-guided purification process of its extract that led to the identification of ergosterol-5,8-peroxide 2 (MIC = 0.78 μg/mL) as responsible for its bioactivity. Previously, this compound 2 has been reported as deriving from the medicinal plant *Ajuga remota* (MIC = 1 μg/mL) (20).

There are no reports, to our knowledge, of compounds isolated from the *Gliocladium* genus with antitubercular activity; however, the fungi of other genera belonging to the Hypocreales order of the Moniliaceae family such as *Paecilomyces tenuipes* produced beauvericin 

(MIC = 12.5 μg/mL) (21). 

This is the first report, to our knowledge, of polyols 3 and 4 from this fungus. Allitol (4) is an acyclic and rare sugar possessing a low abundance in nature and with an exorbitant cost (22). It was previously isolated from the fungus *Tylopilus plumbeoviolaceus *(23). No reports exist, to our knowledge, for allitol (4) with respect to its biological activity in the scientific literature; however, its isomeric forms, such as mannitol, have been reported to possess activity against *Bacillus subtilis*, *Escherichia coli*, and *Staphylococcus aureus *(24). Our research contributes to the chemical composition and antitubercular activity of the fungus *Gliocladium *sp. MR41.
